# Investigation of the roles of microglia and astrocytes in cerebral amyloid angiopathy across diverse murine strains

**DOI:** 10.1002/alz70855_101282

**Published:** 2025-12-23

**Authors:** Jonathan Nyandu Kanyinda, Olivia J Marola, Gareth R Howell

**Affiliations:** ^1^ The Jackson Laboratory, Bar Harbor, ME, USA; ^2^ University of Maine, Orono, ME, USA; ^3^ Tufts University Graduate School of Biomedical Sciences, Boston, MA, USA

## Abstract

**Background:**

Cerebral amyloid angiopathy (CAA) is the accumulation of amyloid beta (Aβ) in the walls of the cerebral vasculature. CAA can cause damage to the vasculature and disrupt proper functioning of the blood brain barrier (BBB). CAA has also been associated with cerebral microbleeds, cognitive decline, and dementia. The roles of the brain's resident immune cells (microglia and astrocytes) in preventing or driving CAA are not fully understood. Microglia and astrocytes are important in the clearance of Aβ through the glymphatic system. Additionally, depletion of microglia has been shown to lessen parenchymal plaque deposition but increase CAA in models of AD, suggesting a potentially protective role for microglia against CAA development.

**Methods:**

Mice with diverse genetic backgrounds (B6, WSB, and BXW) expressing *APP/PS1* were utilized to understand genetic drivers of CAA development. Brains were evaluated at 8M. CAA was assessed using immunohistochemistry (X34+ plaques) within arteries (αSMA + vessels). CAA area, plaque area, IBA1+ area, and GFAP+ area of 8M brains (*n* = 3) were quantified using custom FIJI scripts that employed standardized thresholding settings. A quantitative analysis was performed using GraphPad Prism to assess the differences between the different genetic backgrounds. Brains at 12M are currently undergoing analysis.

**Results:**

We have shown that B6 and WSB genetic backgrounds are less susceptible to CAA compared to BXW, which develops robust CAA. Preliminary analysis indicates WSB brains have fewer but larger plaques than B6 and BXW. WSB mice also had less GFAP+ area per plaque compared to B6 and BXW mice. In contrast, BXW mice had more IBA+ area per plaque compared to B6 and WSB mice.

**Conclusions:**

Given the differential susceptibility of B6, WSB, and BXW genetic backgrounds to CAA development and the potential role of microglia and astrocytes in modulating susceptibility to CAA. It is possible that B6, WSB and BXW.*APP/PS1*'s unique cell response influences amyloid deposition in the vasculature, leading to CAA. Understanding the role of microglia and astrocytes in AD‐relevant pathologies will help further the field in the target microglial activity and proliferation as it shows an inverse relationship with CAA expression across strains.

